# Neurologic Assessment of the Neurocritical Care Patient

**DOI:** 10.3389/fneur.2021.588989

**Published:** 2021-03-22

**Authors:** Shane Musick, Anthony Alberico

**Affiliations:** Department of Neurosurgery, Joan C. Edwards School of Medicine, Marshall University, Huntington, WV, United States

**Keywords:** neurological wake-up test, multimodality monitoring, neurologic examination, daily-interruption of sedation, traumatic brain injury, sedation cessation

## Abstract

Sedation is a ubiquitous practice in ICUs and NCCUs. It has the benefit of reducing cerebral energy demands, but also precludes an accurate neurologic assessment. Because of this, sedation is intermittently stopped for the purposes of a neurologic assessment, which is termed a neurologic wake-up test (NWT). NWTs are considered to be the gold-standard in continued assessment of brain-injured patients under sedation. NWTs also produce an acute stress response that is accompanied by elevations in blood pressure, respiratory rate, heart rate, and ICP. Utilization of cerebral microdialysis and brain tissue oxygen monitoring in small cohorts of brain-injured patients suggests that this is not mirrored by alterations in cerebral metabolism, and seldom affects oxygenation. The hard contraindications for the NWT are preexisting intracranial hypertension, barbiturate treatment, status epilepticus, and hyperthermia. However, hemodynamic instability, sedative use for primary ICP control, and sedative use for severe agitation or respiratory distress are considered significant safety concerns. Despite ubiquitous recommendation, it is not clear if additional clinically relevant information is gleaned through its use, especially with the contemporaneous utilization of multimodality monitoring. Various monitoring modalities provide unique and pertinent information about neurologic function, however, their role in improving patient outcomes and guiding treatment plans has not been fully elucidated. There is a paucity of information pertaining to the optimal frequency of NWTs, and if it differs based on type of injury. Only one concrete recommendation was found in the literature, exemplifying the uncertainty surrounding its utility. The most common sedative used and recommended is propofol because of its rapid onset, short duration, and reduction of cerebral energy requirements. Dexmedetomidine may be employed to facilitate serial NWTs, and should always be used in the non-intubated patient or if propofol infusion syndrome (PRIS) develops. Midazolam is not recommended due to tissue accumulation and residual sedation confounding a reliable NWT. Thus, NWTs are well-tolerated in selected patients and remain recommended as the gold-standard for continued neuromonitoring. Predicated upon one expert panel, they should be performed at least one time per day. Propofol or dexmedetomidine are the main sedative choices, both enabling a rapid awakening and consistent NWT.

## Introduction

There is widespread use of sedation for patients in the intensive care unit (ICU) and neurocritical care unit (NCCU). This is a necessary practice to facilitate endotracheal intubation and mechanical ventilation, however, it is also Janus-faced. There is good clinical utility, such as controlling patient distress, attenuating anxiety, and abating pain recognition (1), with neuro-specific benefits including reduced metabolic demands to decrease energy consumption (2), decreased stress-related injury, as well as seizure, temperature, and intracranial pressure (ICP) control (3, 4). However, over sedation harbors complications, including increased morbidity (5, 6), prolonged ventilation with associated pneumonia (3), greater muscular atrophy, venous stasis, thrombosis, and a protracted ICU length of stay. Further, it may increase hospital costs secondary to ordering unnecessary neuroimaging (1). Too little sedation can magnify agitation and autonomic instability, leading to elevated ICP, hypertension, tachycardia, and cerebral oxygen consumption (1). Thus, risks and benefits must be carefully weighed when it comes to achieving optimal sedation.

Sedation also precludes an accurate neurologic examination, and continued sedation may mask significant changes in the patient's neurologic condition (7). This is concerning, as upwards of 40% of patients with a traumatic brain injury (TBI) demonstrate a significant deterioration of neurologic function within 10 days (8). There is a known secondary deterioration that evolves during the early period of brain injury that is heterogenous and hard to predict (9). This is due to secondary injury cascades that activate inflammatory, excitotoxic, metabolic, and vascular phenomena. This augments oxidative stress, elevates ICP and metabolic demands, causes cerebral edema, activates coagulation cascades, and impairs regional blood flow (7, 10). Continued sedation can also prevent the acquisition of an accurate Glasgow coma scale (GCS) score. This is imperative, as GCS scores are a robust prognostic marker and indicator of potential surgical intervention (11, 12), and are highly predictive of 6-month outcomes in TBI patients (13). This underlies the necessity for a brief cessation of sedation for an accurate neurologic assessment, termed the neurological wake-up test (NWT). The NWT is considered to be the gold-standard for neuro-monitoring (1, 3), and is the basis for neuroanatomical localization of pathology, identifying undiagnosed neurologic ailments, detecting early neurologic signs of insult, determining prognosis, and guiding appropriate therapy (3, 7, 14).

Serial NWTs are an integral part of continued ICU and NCCU assessment of neurologic functioning but are concerning because they require a temporary cessation of sedation. This results in a significant sympathetic nervous system (SNS) discharge, potentially resulting in neurologic injury via elevating ICP, increasing cerebral oxygen demand, and reducing cerebral perfusion. However, this must be weighed against the additional clinical information acquired. Moreover, with the increasing utilization of multimodality monitoring, there may be enough information gathered to make the NWT both harmful and redundant. Therefore, this paper will aim to elucidate the utility of the NWT, if it still has a role with multimodality monitoring, if there is an optimal frequency that imparts the most favorable risk to benefit profile, and if the choice of sedative has an influence on these quandaries. For this review, PubMed was searched for all existing literature using the terms brain injury, head injury, or TBI, with the terms sedation cessation, daily interruption of sedation, wake-up test, stopping sedation, spontaneous awakening trial, neurologic examination, multimodality monitoring, frequency of awakening, and/or frequency of neurologic exam.

## Multimodality Neuromonitoring

A healthy brain has robust autoregulatory mechanisms to maintain constant cerebral blood flow (CBF) across a range of mean arterial pressures (MAPs) from 65 to 150 mmHg. In the presence of neurologic insult, there is often regional or global impairment of autoregulation (1). Thus, continued monitoring of ICP and CPP are often necessary. Utilizing transcranial doppler (TCD) may assist in assessing the degree of autoregulation failure (15). Other monitoring modalities include brain tissue oxygen tension monitoring (PbtiO_2_), jugular venous oxygen saturation (SjvO_2_), and brain neurochemistry by intracerebral microdialysis (MD). Newer, less invasive monitoring technologies including optic ultrasound, and the automated pupillometer to directly augment the NWT are also seeing increased use. Lastly, a role for brain injury biomarkers in diagnosis and prognosis is beginning to emerge.

### ICP

Normal ICP values are between 5 and 15 mmHg (16). Increased ICP is defined as pressures >20 mmHg for more than 5 min. This monitoring can be achieved through insertion of a catheter into the lateral ventricle to act as an external ventricular drain (EVD), or via insertion of an intraparenchymal monitor (IPM) (7). There is frequent discordance in EVD vs. IPM measures, but little consistency exists about over vs. underestimating to allow for correction (17). The EVD measure is considered most accurate, but it also harbors increased rates of infection and hemorrhage. Furthermore, when maintained in a continuous open state, EVD measures are often erroneous (18). EVD has been suggested for use due to its ability to drain CSF, which can aid in controlling refractory ICP elevations (19).

ICP monitoring is recommended as part of the official TBI guidelines (20); consequently, there is widespread use of ICP monitoring of brain injured patients across NCCUs (7, 9, 21). The Brain Trauma Foundation (BTF) recommends monitoring for comatose patients [GCS of (3–8)] that have an abnormal CT scan (22). Based upon the International Multidisciplinary Consensus Conference on multimodality monitoring, ICP monitoring is strongly recommend, along with clinical examinations and other monitoring modalities to accurately prognosticate and guide treatment (11). ICP is frequently elevated following neurologic insult, and is a recognized cause of morbidity and especially mortality after TBI (7, 22–25). There is evidence to suggest that aggressive management of elevated ICP can improve outcomes in TBI patients (9, 22). Conversely, concerns have been raised regarding no improved clinical outcomes with ICP monitoring, and possibly increased mortality rates from its use, at least in TBI patients (7, 26). This was corroborated by the BEST-TRIP trial (27), which demonstrated that treatment guided by ICP-monitoring was not superior to treatment guided by the NWT and serial CT scans. In patients with hemispheric ischemic stroke and associated cerebral edema, ICP monitoring is not recommended (28).

### Optic Ultrasound

Optic ultrasound is a quick, cost-effective, non-invasive method to measure ICP by evaluating optic nerve sheath diameter (ONSD). The optic nerve sheath is contiguous with the dura and contains CSF that communicates with cerebral subarachnoid components (29). Measuring 3-mm behind the globe at the anterior portion of the optic nerve, an ONSD of 5-mm translates to an ICP of ~20 mmHg (29). A meta-analysis of six studies calculated a sensitivity of 90%, and specificity of 85%, for detecting elevated ICP (30). Another prospective study calculated a sensitivity of 93.75%, and specificity of 86.67% for identifying increased ICP (31). This means that somewhere between 6 and 10% of patients with elevated ICP will be missed using this monitoring modality. This may be an acceptable trade-off given its quick bedside ease of use, and when coupled with clinical exam findings, may offset those limitations. Overall, measuring ONSD has established itself as a reliable initial screening tool for detecting ICP changes (32). It can also be useful to track individual changes quickly per patient, as ONSD changes occur within 5 min of ICP shifts (33). This may play an especially crucial role in situations where access to invasive monitoring techniques are unavailable.

### CPP

rCPP can be calculated with ICP monitoring, as CPP = MAP – ICP. Uncertainty exists surrounding differing placements of zero reference points for ICP and MAP, which represents a technical hurdle in attaining accurate and consistent CPP values. This is a major issue, as many of the studies behind recommended CPP thresholds did not report their methods for obtaining CPP (34, 35). Moreover, the BTF guidelines state that CPP is calibrated to the level of the right atrium by convention (20). Most brain injured patients are maintained at 30° of head elevation, and the resultant 30 cm distance between the heart and head can overestimate CPP by up to 11 mmHg, and at elevations up to 50°, the CPP can be overestimated by 18 mmHg (36). Thus, in patients with head of the bed elevation, it is crucial that the arterial transducer is positioned at the level of the middle cranial fossa, which is approximated to the tragus, to ensure accurate CPP measurements (37).

The BTF recommends a CPP range between 60 and 70 mmHg (20). Crucially, CPP elevation past 70 mmHg has been linked to poor outcomes in TBI patients, along with lung damage, whereas CPP levels below 70 mmHg have been associated with worsening brain hypoxia (7). Andrews and colleagues found that low CPP along with hypotension was the best predictor of death in TBI patients (38). However, there is great variability in outcomes. In one study half of the patients benefited from higher CPP, and the other half benefited from lower CPP (39). To attenuate this variability, some recommend individualizing the CPP target (24, 40, 41) through the use of cerebral autoregulation monitoring (42).

### SjvO_2_

SjvO_2_ monitoring is used to acquire information pertaining to cerebral oxygen supply, perfusion, and consumption. It is performed via fiberoptic catheters placed into the internal jugular bulb distal to the jugular foramen, or intermittently checking jugular venous blood samples (7, 9). Falsely elevated SjvO_2_ can be measured due to the presence of only extracerebral blood in the jugular bulb (43), which depends on contamination from extracerebral sources via aspirating too quickly, or misplacing the catheter by a couple of centimeters (44). This modality is of limited utility in severe global ischemia or very large infarcts, as SjvO_2_ can rebound upward due incomplete oxygen extraction by the ischemic tissue (43, 45).

Normal values range from 55 to 75% oxygen saturation (O_2_ sat). Lower values are indicative of ischemia, and values <50 and > 75% are both associated with poor patient outcomes (7, 9, 45). However, there are concerns about the clinical utility of this measurement, as one positron emission tomography (PET) study demonstrated that the SjvO_2_ value did not drop to <50% until ~13% of the brain became ischemic (46). Normal range SjvO_2_ values are also often obtained in the presence of ongoing focal ischemia, hyperemia, and/or shunting (9), along with frequent false positive desaturations (9, 47). Vidgeon et al. state that there is no solid evidence to support its use for ongoing clinical monitoring (9).

### PbtiO_2_

Brain tissue oxygenation monitoring provides information pertaining to focal oxygenation, with typical values ranging from 15 to 30 mmHg (2–4 kPa), and a critical hypoxic threshold commonly established at 10 mmHg (<1.33 kPa) (7, 9). This monitoring is carried out through a thin electrode placed in either peri-ischemic at-risk tissue for focal measurements, or in frontal white matter to estimate global cerebral oxygenation in diffuse brain injury (7, 9). Ischemic changes with regional differences have been detected following TBI (48), and these transient periods of ischemia are correlated with worsened patient outcomes (49). Brain injured patients with brain hypoxia (PbtiO_2_ <10 mmHg) have significantly poorer outcomes and increased mortality (50). The BOOST-II trial demonstrated improved outcomes with less mortality when guiding therapy by PbtiO_2_ plus ICP monitoring vs. ICP alone (51). A systematic review comparing PbtiO_2_-based therapy alongside ICP/CPP monitoring to ICP/CPP-based therapy alone reported a favorable outcome for the PbtiO_2_-based group (52). Not all trials report a positive outcome, and they are largely based on low quality of evidence, therefore PbtiO_2_-guided therapy and clinical outcomes remain subject to debate (53).

### Intracerebral Microdialysis (MD)

MD is utilized to measure brain neurochemistry. It is performed via the insertion of a microdialysis catheter that contains a semipermeable membrane, which is perfused with artificial CSF, allowing passive diffusion and measurement of various neurotransmitters, and metabolic substrates and products, like glucose, lactate, pyruvate, glycerol, glutamate, et cetera (7). The MD catheter may be placed adjacent to a focal lesion to detect early metabolic alterations, or in the non-dominant frontal region in the case of diffuse injury (9, 54). Its most promising application is detecting ischemia and neuronal damage prior to being clinically detectable, allowing for early intervention to salvage brain tissue (40, 55). LPR is a sensitive marker of brain ischemia and redox state (9, 54). Elevated LPR measurements correlate with symptom severity and fatal outcomes after brain injury (54, 55). Elevated LPR > 25 is associated with poor outcomes in TBI (9), and elevated LRP coupled with low glucose correlates with worsened outcomes in TBI and subarachnoid hemorrhage (SAH) patients (40, 56). One study showed that length of time spent with elevated LPR > 40 correlated with frontal lobe atrophy at 6 months (57). Thus, MD offers unique insight into cellular bioenergetics and their perturbations following brain injury. There is increasing use of MD, and certain protocols have been established, with alarm levels of LPR set at >30, and/or glucose levels <0.8 mmol/l (58). Despite the promising utility, its overall value as a tool for guiding clinical decision making has yet to be fully elucidated (7, 40, 58).

### Regional CBF

Thermal diffusion flowmetry (TDF) can directly measure regional CBF (rCBF). This measurement is achieved through insertion of a probe into the brain with proximal and distal thermistors, which calculates the power required to maintain a temperature difference between them, and this is proportional to cerebral tissue perfusion (59). It is typically inserted nearby “at-risk” tissue, such as the white matter of a vascular territory at risk for vasospasm (60). Its use is limited due to sparse clinical data on how it can guide management, its ability to measure only a very small volume, and its high sensitivity to positioning (60, 61). Consistently, the consensus summary on multimodality monitoring state that “a TDF probe may be used to identify patients with focal ischemic risk within the vascular territory of the probe,” citing a weak recommendation with very low quality evidence (11). A recent systematic review found that both very low and very high rCBF measures were associated with poor outcome and correlated with intracranial hypertension, and that rCBF and PbtiO_2_ were largely congruent (59). However, they stress the lack of data, stating much more research needs to be undertaken prior to widespread adoption.

### Brain Injury Biomarkers

Measurement of blood or CSF brain injury biomarkers is a cost effective and less invasive tool that may assist in triaging, prognosticating, and following the course of disease. The most studied of these include S100B, glial fibrillary acidic protein (GFAP), ubiquitin carboxyl-terminal hydrolase isozyme L1 (UCH-L1), neuron-specific enolase (NSE), and neurofilament light chain (NFL). Among these, only S100B is part of an official set of TBI guidelines where it has suboptimal real-world performance (62), largely owing to poor specificity and large numbers of false positives (63, 64). In those guidelines, it is utilized to triage emergency department (ED) patients for CT scans based on S100B levels (65). In the ALERT-TBI study, they found a combination of GFAP and UCH-L1 was highly sensitive in triaging patients for CT scans (66). In another report, GFAP levels were highly predictive of CT positivity, and adding other biomarkers did not improve discrimination (62). Accordingly, an active role for biomarker measures in continued NCCU assessment and guiding therapies is tenuous, because rather than portending impending secondary insults, late elevations signify the occurrence of secondary damage (65).

Nevertheless, the various biomarkers are still robust markers of neuronal damage. Increased S100B levels within 24 h of severe TBI strongly correlates with mortality, as does elevated NSE and GFAP (67), while also correlating with lesion expansion and brain hypoxia (65). Therefore, serial measures in the NCCU can aid in establishing extent of damage, or if new damage is ongoing. This can be refined by taking differing kinetics into account, such as GFAP vs. S100B, whereas the former persists for days after an initial injury, the latter rises and falls within hours (68). The prognostic and diagnostic impact of such measures is obvious, but how they can guide continued management remains unclear. Furthermore, given their reliability as markers of neuronal damage, research utilizing biomarkers in patients undergoing NWTs will be an important step in its continued safety assessment.

### Combination Monitoring

Each of these multimodal monitoring components provide unique and clinically relevant information pertaining to neurologic functioning. Multiple modalities can complement the information from one another synergistically, hence the rationale for using multiple monitoring modalities concurrently (9). When used in conjunction, the emergence of similar pathologic patterns can point to the cause of underlying deterioration or increase the likelihood of picking up early changes. It is also imperative to utilize a combination of regional (MD, PbtiO_2_, TDF) and global (ICP, CPP, SjvO_2_, biomarkers) monitoring to ensure a complete picture of ongoing processes.

For instance, Muizelaar, while evaluating the utility of multimodality monitoring to predict hypoperfusion, recommends utilizing a combination of ICP, CPP, and PbtiO_2_ monitoring (69). Aligning with this, Smith et al. state that the simultaneous measurement of ICP and brain tissue oxygenation is a simple, logical approach, as a single probe can monitor both measurements (41). Accordingly, the Seattle International Brain Injury Consensus Conference (SIBICC) recently stated that PbtiO_2_ should be the second monitored variable after ICP (70).

### Multimodality Neuromonitoring Conclusion

Recently, the Neurocritical Care Society and the European Society of Intensive Care Medicine held a panel to evaluate and discuss the evidence of multimodality monitoring (24). They conclude that a single monitoring modality is demonstrably insufficient. Despite this, they state that there is no consensus on their use, and more studies are required to ascertain if utilization translates to improved patient outcomes (24). However, they underscore the importance of the neurologic exam and the NWT, stating that it remains a cornerstone in the accurate assessment of patients. The vast amounts of information gathered via these monitoring modalities, how to evaluate and integrate them, and their ability to guide optimal therapeutic plans is still being elucidated (11). Currently, though multimodality guided therapy can improve neurologic physiological variables, it has yet to show an improvement in outcomes (60).

## The NWT

Temporarily and intermittently stopping sedation of ventilated patients to mitigate the harmful effects of over sedation is termed a daily interruption of continuous sedation (DIS) trial. Though widely utilized and studied, firm recommendations are tenuous because of large scale reviews with conflicting conclusions about their utility (3, 71). Despite lacking firm recommendations for DIS protocols, the NWT, which involves a daily cessation of sedation for neurological examination purposes, is regarded as the gold-standard for evaluating patients with brain injury in the ICU and NCCU (1, 3, 7, 14, 24, 72–74). Neither neuroimaging, nor multimodality monitoring can replace the neurologic examination (75), and it remains the most valuable tool for the assessment of brain injured patients, from stroke (74), to SAH (76, 77), and TBI (14, 24, 72). A recent intensive care symposium in Paris aiming to update neurocritical care recommendations states that the neurologic examination is indispensable for the accurate assessment of comatose patients, along with the simultaneous use of neuroimaging and multimodality monitoring (78). The SIBICC panel recommend a sedation holiday (an NWT) in TBI patients with ongoing ICP monitoring to facilitate an accurate neurologic exam (79). They could not reach consensus on absolute nor relative contraindications, but they do not endorse the NWT until ICP is within acceptable limits (<22 mmHg) for at least 24 h. This is in line with preexisting intracranial hypertension being regarded as an absolute contraindication to the NWT (14). Additional hard contraindications include barbiturate treatment, status epilepticus, or hyperthermia.

Importantly, the NWT is not akin to a true awakening response, but rather an arousal reaction (73). Essential components of the NWT include GCS rating of the motor component (GCS-M), by asking the patient to obey simple commands, such as moving an extremity, squeezing the practitioner's fingers, etc. If no responses are elicited, a painful stimulus is provided, such as a sternal rub, supraorbital pressure, mandibular pressure, or a trapezius squeeze, and the provoked motor response is recorded. The other essential components of the NWT include evaluation of pupil diameter with attention paid to anisocoria, both direct and indirect pupillary light reflexes, and any focal neurologic deficits along each extremity (3). The automated infrared pupillometer has emerged as a rapid, noninvasive neuromonitoring tool to provide an objective assessment of pupillary reactivity, and drastically increase reliability and sensitivity of the pupil examination (41, 80–82). Deterioration of the pupillary light reflex is a strong predictor of outcome after brain injury, and subtle pupillary changes are often a harbinger of elevated ICP, secondary brain injury, cerebral edema, hydrocephalus, and intracranial shift (80).

Neuroworsening, defined as a reduction in GCS-M ≥ 2 points, or development of pupillary anomalies, mandates further investigation (83). Irrespective of the utility of multimodality monitoring, the NWT remains integral in the overall evaluation of patients. Evolving pathology can be picked up earlier, and some deterioration may only be detected by physical examination (7). This helps in patient assessment, and in monitoring treatment effectiveness (24). Many patients may have ongoing damage without clear abnormalities picked up through other monitoring modalities (83). The NWT is especially useful following decompressive craniectomy, or cases of SAH-related vasospasm, as deterioration may occur before ICP elevations and neuroimaging changes are detected (76). In cases of temporal hematomas, brain herniation can occur without concomitant elevations in ICP (16, 84). In patients with cerebral edema secondary to hemispheric ischemic stroke, such as malignant middle cerebral artery (MCA) infarction, ICP monitoring is not recommended because ICP elevations do not occur for days, and midline shift and pupillary abnormalities often occur even with ICP values <20 mmHg (28, 74, 85). These cases necessitate NWTs for early detection. This may become more pertinent with the 2020 BTF update recommending decompressive craniectomy for control of late, medically refractory ICP elevation (86). Table 1 outlines the indications and contraindications.

**Table 1:** Brief outline of indications and contraindications to the NWT.

**Indications**	**Contraindications (3)**
When CT is unimpressive/borderline in a patient who has been sedated and intubated prior to imaging (65)	Preexisting intracranial hypertension (ICP > 20 mmHg for > 5 min)
Patients with SAH – to detect vasospasm (76)	Ongoing or recent barbiturate treatment
Patients with temporal hematomas or following decompressive craniectomy (3)	Status epilepticus
In general, for clinical monitoring in sedated and intubated patients devoid of contraindications, myriad significant safety concerns, or substantial bodily injuries	Hyperthermia (≥38.0–38.5°C)
Evaluation following successful surgical removal of intracranial masses (10)	Patient has reduced intracranial compliance and develops volatile ICP/CPP reactions in response to NWT (87)

### NWT Safety

Despite presumed benefit provided by NWTs, some concerns have been raised pertaining to the acute stress response elicited with discontinuation of sedation. During each arousal, there is a significant acute stress response with SNS discharge that induces hypertension and tachycardia (73). This is reflected by significantly increased levels of stress hormones. Adrenocorticotrophic hormone (ACTH) can be increased by 72.5%, cortisol by 30.7%, epinephrine (E) by 87.5%, and norepinephrine (NE) by 40.4% (88). However, the increased levels of NE do not reach levels required for augmenting the risk of microthrombi formation (89). These changes are mirrored by significantly increased ICP and CPP values, with a mean increase of 3 and 8 mmHg, respectively. During the NWT, ICP reached an average value of 15.3 mmHg, and CPP reached an average value of 84.4 mmHg (88).

Skoglund et al. explored ICP and CPP changes in TBI and SAH patients (21 patients) undergoing NWTs (127 total NWTs). In pooling all patients, they demonstrated a mean increase in ICP of 8 mmHg, reaching an average value of > 20 mmHg. The mean CPP increase was 5.2 mmHg, reaching average values of > 81 mmHg (76). There were important subgroup differences, with TBI patients showing higher ICP values at baseline and during the NWT, and SAH patients presenting with higher CPP values at baseline and greater changes during the NWT, indicating the need for patient stratification based upon injury type and baseline characteristics. Furthermore, highlighting the heterogeneity of brain injuries, there was great variability in these metrics. Several patients developed a sustained increase in ICP levels to >30 mmHg for an average of 3.6 min. Similarly, some patients had a sustained decrease in CPP values to ≤ 50 mmHg for an average of 10 min, and these reductions were far more common in TBI patients. Overall, in 23/127 trials, CPP decreased to <50 mmHg due to ICP ≥ 25 mmHg, although these were generally short-lived deviations. The average ICP values peaking at >20 mmHg were interpreted to be safe and well-tolerated by the authors due to their mild and transient nature. Consistently, they refrained from performing NWTs in clearly unstable patients and those with plateau waves, which are sudden rapid elevations of ICP to 50–100 mmHg due to cerebral vasodilation (90). In contrast to the current study (76), the previously discussed research by Skoglund and colleagues showed less dramatic ICP and CPP elevations in response to the NWT (88). Stover hypothesizes that these discrepancies may reflect a learning curve to the NWT, or involve examinations undertaken on less severely injured patients with preserved autoregulation (73). In either case, it certainly indicates the heterogeneity of brain injury and the unpredictable clinical course that may ensue (9).

Excluding those with preexisting intracranial hypertension, the increases in ICP and CCP were transient and interpreted to be well-tolerated without advancing neurologic deterioration (3). Additionally, though these increases occur, they are not met with alterations in overall cerebral metabolism or oxygenation, suggesting its safety in most patients (1, 24). Components of multimodality monitoring were evaluated for changes occurring due to the NWT in 17 severe TBI patients (11 focal, 6 diffuse/mixed) (91). Patients were included if they had a severe TBI and were mechanically ventilated under propofol sedation, with ongoing MD, PbtiO_2_, and/or SjvO_2_ monitoring. They were excluded if they received recent or ongoing barbiturate infusion, required continuous propofol sedation for ICP control or tube tolerance, or if they were unstable. It was demonstrated that there were no significant changes in measures of glucose, lactate, pyruvate, glutamate, glycerol, or LPR as measured by MD. Similarly, changes in oxygenation as measured by both PbtiO_2_ and SjvO_2_ showed no significant alterations. Consistent with previous reports, ICP and CPP values significantly increased during the NWT, by 7.6 and 6 mmHg, respectively, reaching values of 16.7 and 94.4 mmHg. These reports demonstrate that although there is a stress response elicited by the NWT, and ICP and CPP elevations occur in parallel, neurochemical and cerebral perfusion alterations are minimal. This indicates that these perturbations are well-tolerated in this cohort of TBI patients, and are unlikely to cause secondary injury to the brain (7, 72).

In one prospective study of 20 patients (54 total NWTs), comprising 12 with TBI and nine with SAH, upwards of 34% of planned NWTs were not attempted as the patients were not seen as stable enough due to elevated ICP, hemodynamic instability, and need for continuous sedation (92). Of the 54 attempted NWTs, upwards of 33% were terminated early due primarily to ICP-crisis (>20 mmHg), agitation (22%), or systemic desaturation (11%). Moreover, in those patients with NWT cessation, they noted a statistically significant reduction in PbtiO_2_ measures, decreasing from an average of 28 mmHg to 19 mmHg, which is still well-above hypoxic thresholds (9, 29). Crucially, in none of their patients did PbtiO_2_ fall below 15 mmHg, which is within commonly cited normal ranges (9). While not rising to the level of statistical significance, a trend was noted between baseline lower brain glucose, higher total LPR, and neurologic deterioration from the NWT, which seems to corroborate the benefit of multiple simultaneous modalities of monitoring. Overall, however, there were no significant alterations in lactate, pyruvate, LPR, or glucose in patients undergoing NWTs. The authors speculate that MD is useful for baseline patient discrimination, without adding value during the NWT itself, at least in their cohort. Logically, this statement does not appear sound, as subtle metabolic alterations would necessarily precede injury progression, especially given their perilesional placement, potentially indicating the NWT is safer than presumed. Likewise, as shown by Skoglund et al. (76, 91), transiently increased ICP to >20 mmHg may not be as deleterious as assumed. If they are not coupled with concomitant metabolic indicators of ischemia or inadequate perfusion, it is unlikely that neurologic deterioration is occurring; in no reports on the NWT to date has there been evidence of secondary deterioration (3). Therefore, though patients that developed intracranial hypertension in response to the NWT had stoppage of the examination, the preponderance of data indicate most of these patients were likely to tolerate the exam without promoting neurologic injury. Nevertheless, differential findings in PbtiO_2_ measures between this report by Helbok et al. (92) and the aforementioned one by Skoglund et al. (91), further demonstrate the marked heterogeneity of brain injured patients and need for scrupulous patient stratification (73). An additional confounder between the two reports is that of considerably different sedative use. Helbok et al. (92) utilized a combination of propofol, midazolam, dexmedetomidine, and fentanyl, whereas Skoglund et al. (91) used continuous propofol infusion with intermittent morphine administration. These significant differences make conclusions questionable, however, it may give insight into optimal sedative practices to facilitate successful NWTs.

Additional predictors of NWT failure were shown by Esnault et al. in a large 7-year study at one center, with 242 patients, of whom 96 underwent an NWT (93). Some significant differences were found between patients that the authors felt should vs. should not undergo NWTs. Notably, patients that did not receive NWTs had higher ISS (26 vs. 16) and SAPS 2 scores (46 vs. 38), indicating more severe total body injuries, along with lower GCS scores (6 vs. 7), more shift (50 patients vs. 15), and the presence of more subdural hematomas (86 patients vs. 40). This underscores that in patients with severe comorbid injuries, and with significant shift, the NWT may be regarded as unsafe. Also, in patients with comorbid injuries, their brain injury, albeit severe, may be outweighed by more deleterious and imminent concerns. Nonetheless, this study demonstrated that 39.5% of their patients had discontinuation of NWTs. Of those patients with discontinued NWTs, it was due to neurologic deterioration in 71% of cases, with the remaining 26% due to respiratory distress. Most neurologic deteriorations were due to seeing no improvement in the neurologic exam (32% of stoppages), or intracranial hypertension [(ICP > 20 mmHg for > 5 min) 16% of stoppages]. As previously discussed, other research has demonstrated that these short-lived increases in ICP from the NWT are not met with alterations in cerebral metabolism or subsequent neurologic deterioration, making it likely that these elevations were not clinically relevant (91, 92). Not only that, the majority of premature stoppages were simply due to seeing no improvement in the exam, which does not constitute a safety concern in itself, bolstering the case for the NWT being well-tolerated in most patients in these studied cohorts. However, the significant number of patients displaying no improvement in their clinical exam is important, as a major function of the NWT is to accurately assess neurologic functioning. However, although the examination did not change in those patients from before to after sedation cessation, it may have still played a role in long-term assessment if subtle changes were later recognized, or if the lack of change remained consistent. They also identified two patient cohorts with a significantly increased probability of NWT failure: those with a subdural hematoma > 5 mm thick on first imaging, or initial GCS <5. Finally, they note that the patients that were unable to tolerate an NWT had significantly worse outcomes at 1 year, implicating the NWT as a long-term prognostic tool.

### Does the NWT Provide Clinical Information?

While the NWT is widely used, there is little information about the clinical benefits procured by it in the literature. Theoretical rationale is a good starting point, but is incomplete on its own (72). Stocchetti et al. demonstrated that out of 449 TBI patients, 12.9% were misclassified as having a more severe brain injury due to sedation masking their neurologic functioning, precluding an accurate assessment (94). Compatibly, in the study by Esnault et al., 21% of patients that underwent a NWT were able to be extubated within 48 h (93). Only one randomized controlled trial of 97 patients (38 head-injured patients) on the NWT exists (95), which demonstrated a reduction in the duration of mechanical ventilation by an average of 3.9 days, and decreased ICU stay by 3 days in the head-injury group, albeit neither of these measures were statistically significant. The differences detected in the head-injury only group are stark in comparison to all pooled patients. The authors note that they estimated 45 patients were needed per group to detect significant changes, and the head-injury only subgroup had 21 in the intervention group and 17 in the control. Therefore, given the magnitude of those differences, it likely was not powered properly to achieve statistical significance in that patient cohort and requires further study in a larger patient population. Importantly, the authors did not comment on any relevant neurologic information gathered via this intervention, only noting that it was safe and well-tolerated. There has been only one study directly commenting on pertinent clinical information gained by performing NWTs (92), which showed that it rarely led to accrual of additional clinically relevant information (3, 96). In this study, when utilizing NWTs, they noted an increase in GCS and Full Outline of UnResponsiveness (FOUR) scores in half of their patients but did not comment on any change in treatment modality, nor associated prognosis. Although, that observation is still important, because it indicates a much more accurate neurologic examination and clinical representation was achieved in utilizing the NWT. In only one patient was a new focal neurologic deficit discovered. In that patient, increased brain lactate and decreased glucose was observed hours prior to the NWT, underscoring the utility of multimodality monitoring.

### ICP Time-Dose Interaction

The transient elevations in ICP in response to the NWT is a point of clinical uncertainty. Most data indicate that sustained elevations in ICP, and ICP elevation refractory to medical management are deleterious and associated with worse outcomes (76, 83, 97). Consistently, the 2020 BTF update recommends decompressive craniectomy for ICP control only when prolonged and medically recalcitrant, noting late intervention improves mortality whereas early intervention does not (86, 98). The concept of “ICP dose” is becoming increasingly investigated. It recognizes that ICP thresholds are arbitrary and meaningless without accounting for the time spent at “deleterious” levels, and without consideration for the complex interactions of ICP, cerebral blood flow, cerebral metabolism, and the feedback mechanisms involved in cerebral autoregulation, neurovascular coupling, and CO_2_ reactivity (99). Multiple studies have begun to investigate this, demonstrating an association between poorer clinical outcomes with greater time spent above certain ICP thresholds (100–102). In one of these reports, it was shown that increased ICP time-dose was associated with higher mortality and poor outcomes, but no association was found between episodic ICP elevations (5 min > 20 mmHg) and outcomes (101). Helbok et al. point out that the injured brain is not aware of thresholds, and that even a “normal ICP” does not guarantee adequate cerebral perfusion, as ICP-dependent changes in CPP are dynamic, and the threshold-based approach is an oversimplification of a complex pathophysiological process (99). In patients with borderline ICP values, they can be stratified and managed appropriately with the addition of clinical examinations and other neuromonitoring to determine the presence or absence of brain hypoxia, cerebral hypoperfusion, or metabolic distress, allowing an individualized approach (99). This concept lends more credence toward the NWT and its associated transient ICP excursions as safe and well-tolerated in a majority of patients.

### NWT Conclusion

The NWT remains contemporaneously regarded as the *sine qua non* for optimal assessment of the brain-injured patient (3, 7, 14, 24, 83, 92). The limited amount of published literature suggests safety and tolerability in most patients, including those with ICP elevations during the NWT itself. At present, patients with pre-existing sustained intracranial hypertension, those with status epilepticus, marked hyperthermia, or undergoing barbiturate treatment have absolute contraindications to the NWT. Other indicators that a patient may be unable to tolerate an NWT and require careful risk stratification include: hemodynamic instability, recent myocardial ischemia, patients with reduced intracranial compliance, ongoing midazolam sedation, or ongoing sedation for the purposes of controlling agitation, tube control, respiratory distress, seizure activity, or as a primary treatment for ICP control. Further predictors of an inability to tolerate an NWT may include: subdural hematoma > 5 mm thick on first imaging or initial GCS <5 (93), or emergence during the examination of shivering, cardiac arrythmias, or systemic desaturation (92). Individual patients with volatile ICP or CPP responses during exam must be handled in a case-by-case basis with appropriate risk-benefit profiles appraised, with more careful appraisal using concurrent multimodality monitoring.

The reliability of the neurologic exam, its cost, and its ability to detect subtle deficits not picked up via multimodality monitoring make it indispensable. Multimodality monitoring can assist in ensuring cerebral metabolism and perfusion/oxygenation are adequate against the background of elevated ICP or a stable exam. This monitoring may be essential in those patients in whom NWTs are contraindicated. It may assist in individualizing treatment, and detecting metabolic distress before injury has completed. In one randomized controlled trial, ICP-guided treatment did not have better outcomes than clinical examination and neuroimaging alone (27). Overall, multimodality neuromonitoring should be seen as a complement to the NWT, and vice versa (41). More investigations are required to tease out the absolute clinical utility of the NWT, with specific regard to patient outcomes and management guidance (73). Current pros vs. cons are listed in Table 2.

**Table 2:** Brief outline of a list of pros and cons of the NWT.

**Pros**	**Cons**
Theoretical and widely touted ability to detect changes in neurologic status or pick up new deficits that other avenues cannot	No documented clinical benefit (ie lack of noted change in management or detection of new deficits)
More active clinical involvement may lead to more active management	Acute stress response with ICP and MAP elevations, and variable CPP changes
DIS protocols generally reduced ICU length of stay, ventilator time, and associated pneumonia. May hold for NWT, with limited evidence in single randomized trial	Increased cerebral metabolic demand and oxygen consumption may lead to deterioration in certain patients
Aid in patient stratification due to some patients displaying volatile ICP/CPP reactions, and others easily tolerating sedation cessation	May be contemporaneously redundant with multimodality neuromonitoring providing large amounts of information into neurologic function

## Is There an Optimal Frequency of Performing NWTs?

Another central question pertaining to NWTs is their optimal frequency of utilization. Unfortunately, there are a paucity of studies examining this issue, and no clear guidelines have been set. Furthermore, due to the NWT not being recommended in established TBI care guidelines (20), there is great variability in their utilization and frequency. However, these guidelines have come under scrutiny due to their reliance on absolute thresholds (103). The recent SIBICC recommends a sedation holiday (NWT) to facilitate an accurate neurologic examination in severe TBI patients with ongoing ICP monitoring, but do not mention frequency (79). One report suggests that upwards of 50% of NCCU centers in Scandinavian countries do not utilize NWTs (104), which may partly be explained by differences in sedative use. In those centers utilizing NWTs, the majority use daily, with sometimes twice daily checks (7, 72), and one center utilizes NWTs between 4 and 6 times per day (7).

This demonstrates the enormous variability in both use and frequency of NWTs, necessitating the need for more data and guidelines to be established to guide clinical practice. Indeed, only one official recommendation has been offered, from the European Society for Intensive Care Medicine (ESICM) NeuroIntensive Care Section (NIC) (14). They convened an expert panel and state that a daily, brief interruption of sedation is recommended to facilitate an accurate neurologic examination (an NWT) and improve outcomes, giving it moderate evidence and a strong recommendation. Additionally, they state that brain injured patients, ICU patients, and in general all critically ill patients, shoulder undergo a neurological examination on initial ICU admission, and at least once daily, giving it moderative evidence, and a best practice recommendation. They also note the clear contraindication, as previously stated, with DIS and NWTs not recommended in patients with preexisting intracranial hypertension, assigning it moderate evidence and a strong recommendation (14). Lastly, in their concluding remarks, they note that despite technological advances, the neurologic examination remains a foundation of accurate evaluation and prognostic assessment of neurologic function, pointing out the robust prognostic value of GCS scores and pupillary light responses. Therefore, the only concrete recommendation that has been put forth pertaining to the optimal frequency of NWTs is at minimum once daily. Randomized controlled trials will need to be undertaken utilizing differential NWT frequencies to gain insight into what the optimal frequency is, if different patient populations have an impact on this, and what influence injury type has. Thus, much more research will need to be carried out before concrete recommendations about NWT frequency can be given and guidelines administered.

## Choice of Sedative

Another consideration is sedative selection. There are myriad sedating agents utilized in the ICU and NCCU, namely propofol, benzodiazepines, dexmedetomidine, opioids, and barbiturates. The most common agents utilized in ICUs and NCCUs are propofol and midazolam (7), and these may be used in conjunction with opioids for additional analgesia. Recently, dexmedetomidine has begun to see increasing use, and it represents another attractive option for sedation. They each possess their own risk vs. benefit profiles.

### Propofol

Propofol enjoys ubiquitous ICU and NCCU use owing to several factors, including its neuroprotective effects. It is recommended to use for ICP control in current TBI-care guidelines (20), and it dampens cerebral metabolic oxygen demand (96). It possesses both rapid onset of action, and rapid plasma clearance, which facilitates reliable recovery of consciousness even after prolonged administration and thereby a consistent NWT. In higher doses it can induce burst suppression, which can effectively treat status epilepticus (96). It can abate oxidative stress, making it especially useful to combat the free radical generation in head injury (105). An MD study in TBI patients comparing propofol to midazolam found no significant differences in measures of LPR, glutamate, glycerol, or glucose between the two agents over a 72-h period (106). The doses utilized in that study may have been inadequate, and it was a short-term, small study, therefore more investigations are required to determine if propofol can improve outcomes via mitigating oxidative stress. Two retrospective studies have shown that sedation with propofol decreased mortality in TBI (107) and hemorrhagic stroke patients (108). It also has risks, including depressing effects on myocardial contractility, reductions in MAP, elevation in pancreatic enzymes and pancreatitis, and the development of propofol infusion syndrome (PRIS) (105). Though ICP reductions occur which mediate elevations in CPP, occasionally, MAP can fall to such an extent that CPP decreases, which requires judicious fluid resuscitation and vasopressor use. PRIS is a rare but extremely dangerous adverse effect of propofol infusion. It can lead to multi-organ failure and when suspected requires immediate cessation of propofol. Early warning signs include unexplained lactic acidosis, increasing need for inotropic agents, lipemia, and Brugada-like ECG changes (109). Risk factors for PRIS include large cumulative doses of propofol, young age, innate mitochondrial impairments, low carbohydrate intake, high fat intake (which propofol itself possesses, owing to the lipid formulation), critical illness, and catecholamine infusion (109). Importantly, PRIS is believed to be more common in TBI patients, largely owing to the larger doses often used for ICP control (105). For this reason, continuous propofol infusion should not be infused at a rate > 4 mg/kg/h for > 48 h (105). Propofol is an ideal agent for the NWT, and is widely recommended and utilized. Although rare, PRIS may limit its utility for long-term use in brain injured patients.

### Midazolam

Midazolam has rapid onset with a rapid recovery. It decreases cerebral oxygen demand, although to a lesser extent than propofol, and has only slight influences on ICP levels (96). Despite possessing a short half-life, when infused continuously, its half-life increases due to both high lipid-solubility with associated tissue accumulation, and the presence of active metabolites that may be deleterious (7, 96). Consistently, midazolam use is associated with higher mortality rates in ICUs (110). Its persistent use leads to protracted sedation and prolonged time to awakening, which will confound a consistent NWT (96, 111). It also causes significant respiratory depression and inhibition of the cough reflex, along with issues of tolerance development and significant withdrawal symptoms upon cessation. Its use is also a risk factor for ICU delirium, which is itself associated with worse outcomes (105). Accordingly, its use increases ICU length of stay, and either propofol or dexmedetomidine sedation is preferred to improve clinical outcomes in intubated ICU patients (112). Midazolam is not recommended for use when serial NWTs are desired.

### Dexmedetomidine

Dexmedetomidine is a sympatholytic agent that can achieve excellent sedation without respiratory depression, while possessing anxiolytic and analgesic properties. It has rapid onset of action and elimination, does not accumulate in tissues, has a half-life of 6 min, and a terminal elimination half-life of 2–2.5 h, making it ideal for a reliable NWT (96, 105, 113). It also decreases ICP through reducing CBF, increases CPP, and reduces incidence of delirium (105). Though it often leads to a slight decrement in blood pressure, there may be an initial vasoconstrictive effect due to peripheral smooth muscle α-2 adrenergic receptors that occurs more rapidly than its sedative and sympatholytic effects (114). One report of all patients undergoing mechanical ventilation demonstrated that dexmedetomidine was associated with fewer ventilator-associated events and increased chance of extubation in comparison to midazolam and propofol (115). However, as the patient cohort did not comprise strictly brain-injured, care must be taken to extrapolate those findings to the NCCU. A meta-analysis of eight studies concluded that dexmedetomidine is a safe and efficacious agent in the NCCU (116). In a TBI murine model, dexmedetomidine showed significant neuroprotective effects (117), although human studies are required to corroborate these findings. Given its known role of impeding SNS discharge (118), it may decrease the injury-promoting catecholaminergic influence in TBI (119), and has been shown to decrease plasma cortisol after administration (120). This could make this choice of sedative especially useful in the NWT for lessening the NE and E excursions. In one report of 198 severe TBI patients, dexmedetomidine facilitated the highest mean daily time in target Richmond Agitation-Sedation Scale (RASS) compared to propofol, or dexmedetomidine plus propofol (121). Moreover, in another report of 85 severe TBI patients it was demonstrated that in comparison to propofol or midazolam, dexmedetomidine patients were better able to be aroused and cooperate, suggesting it may facilitate a more appropriate level of sedation, better enabling serial NWTs (122). Overall, this agent represents an extremely attractive option to utilize for long-term sedation and to facilitate an NWT. However, more studies are required, and given its limited clinical data, some authors do not currently recommend its use for sedation in brain-injured patients (96).

### Sedative Conclusion

Most recommendations call for continuous propofol sedation, allowing for more frequent cessation of sedation for NWTs (3, 7, 73, 105). There has been some consideration of maintaining a low-dose of analgesics during NWTs (73). Thus, the few recommendations on the subject suggest using propofol to facilitate a smoother transition toward the NWT, with careful attention paid to the development of PRIS, and ensuring infusion rates remain <4 mg/kg/h unless for bolus ICP control. Dexmedetomidine has not yet received strong recommendations, especially in the NCCU, due to an absence of large-scale data. However, the existing literature indicates that dexmedetomidine can be safely and efficaciously used for brain-injured patients, and its rapid onset and short half-life devoid of residual tissue accumulation make it a very attractive choice to facilitate serial NWTs. It is already utilized in NCCUs that employ NWTs and has established feasibility and tolerability for the process (92). Moreover, a recent retrospective observational study found that dexmedetomidine was more commonly prescribed than propofol to facilitate frequent neurologic assessments (123). It is strongly recommended as the sedative agent of choice in the non-intubated patient or with development of PRIS (105). The primary choice of sedative should be individualized, and further based on comfort levels and experience.

Midazolam use is not recommended for sedation because it precludes the ability to perform reliable, serial NWTs (105); it is associated with greater ICU length of stay, duration of mechanical ventilation, and delirium compared to propofol or dexmedetomidine (124); and is associated with dramatically prolonged time to awakening compared to propofol (125–128) and dexmedetomidine (129, 130). Despite the numerous pitfalls of midazolam use, it remains one of the most common sedatives utilized in ICUs (3). Therefore, in centers that primarily use midazolam, it must be recognized that NWTs may lack consistency due to bioaccumulation and residual sedation, with much greater time to awakening. Notwithstanding, when multimodality monitoring is not utilized, the NWT becomes the only source of information about neurologic function. This is already a reality in many lower-income countries and areas, with assessment coming from neurologic exams and serial CT imaging (131). In those cases, the NWT should still be done despite midazolam use, with acknowledgment that time to awakening is increased and day to day reliability of the exam diminished.

## Conclusion

It is widely recognized that the NWT is considered the gold-standard for continued evaluation of brain-injured patients. Hard contraindications exist for patients with preexisting intracranial hypertension, hyperthermia, in status-epilepticus, or on barbiturate therapy. The NWT also induces a significant systemic stress response with ICP elevations, but there has yet to be evidence of secondary deterioration, plus MD and PbtiO_2_ metrics have shown no alterations in the few reports on the subject. The advent of multimodality neuromonitoring has added a tremendous amount of data pertaining to neurologic function into the physician's armamentarium, but have yet to demonstrate clearly improved patient outcomes, and uncertainties exist about their ability to change and guide clinical decisions. NWT frequency is an additional point of uncertainty; the only available expert recommendation endorsed a frequency of at least once per day. Propofol is the most widely recommended sedative to facilitate serial NWTs, but dexmedetomidine represents another viable choice, especially in the non-intubated patient or should PRIS develop. More research is required to elucidate the clinical utility of the NWT, its ability to detect neurologic insults and guide appropriate management, and establish guidelines about the optimal frequency of its utilization with stratification based on injury type and patient population.

## Author Contributions

SM undertook all of the literature search and task of writing the manuscript. AA, as the senior faculty, provided the idea for the review topic and assisted in editing. All authors contributed to the article and approved the submitted version.

## Conflict of Interest

The authors declare that the research was conducted in the absence of any commercial or financial relationships that could be construed as a potential conflict of interest.

## Abbreviations

ACTH: adrenocorticotrophic hormone
BTF: Brain trauma foundation
CBF: cerebral blood flow
CMRO_2_: cerebral metabolic demand for O_2_
CPP: Cerebral perfusion pressure
CSF: Cerebrospinal fluid
CT: computed tomography
DIS: daily interruption of continuous sedation
E: epinephrine
ED: emergency department
ESICM: European Society for Intensive Care Medicine
EVD: External ventricular drain
FOUR: Full Outline of UnResponsiveness
GCS: Glasgow coma scale
GCS-M: Glasgow coma scale – motor component
GFAP: glial fibrillary acidic protein
ICP: Intracranial pressure
ICU: Intensive care unit
IPM: Intraparenchymal monitors
ISS: injury severity score
LPR: Lactate to pyruvate ratio
MAP: Mean arterial pressure
MCA: middle cerebral artery
MD: Intracerebral microdialysis
NCCU: Neurocritical care unit
NE: norepinephrine
NFL: Neurofilament light chain
NIC: NeuroIntensive Care Section
NSE: neuron-specific enolase
NWT: Neurological wake-up test
O_2_ sat: oxygen saturation
ONSD: Optic nerve sheath diameter
PbtiO_2_: brain tissue oxygen tension
PDH: pyruvate dehydrogenase
PET: positron emission tomography
PRIS: Propofol infusion syndrome
rCBF: regional cerebral blood flow
SAH: subarachnoid hemorrhage
SAPS 2: Simplified acute physiology score
SIBICC: Seattle International Brain Injury Consensus Conference
SjvO_2_: jugular venous oxygen saturation
SNS: Sympathetic nervous system
TBI: Traumatic brain injury
TCD: Transcranial doppler
TDF: Thermal diffusion flowmetry
UCH-L1: ubiquitin carboxyl-terminal hydrolase isozyme L1.
